# How do surgeons make decisions about referral to oncology services?

**DOI:** 10.1186/1472-6963-14-S2-P131

**Published:** 2014-07-07

**Authors:** Robin Urquhart, Cynthia Kendell, Joan Sargeant, Gordon Buduhan, Daniel Rayson, Paul Johnson, Eva Grunfeld, Geoffrey Porter

**Affiliations:** 1 Department of Surgery, Dalhousie University, Halifax, Nova Scotia, Canada; 2 Division of Medical Education, Dalhousie University, Halifax, Nova Scotia, Canada; 3 Department of Surgery, University of Manitoba, Winnipeg, Manitoba, Canada; 4 Division of Medical Oncology, Dalhousie University, Halifax, Nova Scotia, Canada; 5 Department of Family and Community Medicine, University of Toronto, Toronto, Ontario, Canada

## Background

Our prior population-based research in Nova Scotia, Canada, demonstrated ‘gaps’ in referral practices for oncology patients with potentially curable disease. The objectives of this study were to examine surgeon decision-making related to referral to oncology services for potentially curable non-small-cell lung, breast, or colorectal cancer patients, and to identify the specific factors that influence their decision to refer (or not to refer).

## Materials and methods

A qualitative study was conducted, guided by the principles of grounded theory. The study design was informed by our ongoing research, as well as a model of access to health services. Data were collected through in-depth, semi-structured interviews with lung, breast, and/or colorectal cancer surgeons in Nova Scotia, Canada (n=28). Data were collected and analyzed concurrently, with two investigators coding and analyzing the data. Analysis involved an inductive, grounded approach using constant comparative analysis. Research team meetings were held to discuss preliminary findings and question the data and interpretations. Data collection and analysis continued until theoretical saturation was reached.

## Results

Seven factors were found to influence surgeon decisionmaking related to oncology referral, with the magnitude of influence differing depending on their decisional proximity. At the core of surgeon decision-making is the clinical encounter wherein the decision is made. Within this encounter, surgeons consider and negotiate their understanding of (1) indications/contraindications for (neo)adjuvant therapy (e.g., tumor pathology, patient comorbidities/health status) alongside (2) patient beliefs and preferences (e.g., the desire or not for chemotherapy). Surrounding the clinical encounter is a number of important mediating factors: (3) a belief that oncologists are the experts, (4) knowledge of local standards of care, and (5) consultation with oncology colleagues. When making decisions about oncology referral, surgeons were also acutely aware of the outer context in which these decisions occur, including (6) system resources and capacity (e.g., access to staging investigations, technology to facilitate coordination of care) and (7) a need to navigate patient logistics (e.g., drug coverage, transportation/lodging). While factors within this outer context infrequently influence referral decisions in a direct way, they often make dealing with the decision more difficult.

## Conclusions

The findings of this study contribute to our broader understanding of how surgeons make decisions about oncology referral and provide a basis to design contextually-appropriate strategies to narrow this gap in our province.

